# Social anxiety and loneliness among older adults: a moderated mediation model

**DOI:** 10.1186/s12889-024-17795-5

**Published:** 2024-02-15

**Authors:** Shuting Sun, Yawen Wang, Lilu Wang, Jinjin Lu, Huihui Li, Jiahui Zhu, Suzhen Qian, Lianlian Zhu, Hongbo Xu

**Affiliations:** 1 School of Nursing, Wenzhou Medical University, University Town, Wenzhou, China; 2 Department of Nursing, Jiangsu Provincial Xuzhou Pharmaceutical Vocational College, Xuzhou, China; 3 School of Public Health and Management, Wenzhou Medical University, Wenzhou, China

**Keywords:** Social anxiety, Social network, Perceived social support, Loneliness, Older adults

## Abstract

**Background:**

Few studies have clarified the mechanisms linking social anxiety and loneliness in older populations. The study aimed to explore how social network mediate the relationship between social anxiety and loneliness in older adults, with perceived social support playing a moderating role.

**Methods:**

A total of 454 older patients completed the Social Avoidance and Distress Scale, Lubben Social Network Scale-6, Chinese version of the Short Loneliness Scale and Perceived Social Support Scale. Bootstrap and simple slope methods were used to test the moderated mediation model.

**Results:**

Social anxiety had a significant positive predictive effect on loneliness and social network partially mediated this relationship. The relationship between social anxiety and social network, as well as the relationship between social network and loneliness, was moderated by perceived social support. Specifically, perceived social support buffered the effects of social anxiety on social network, but the buffering effect diminished with increasing levels of social anxiety. On the social network and loneliness pathway, the social network of older persons with higher perceived social support has a stronger prediction of loneliness.

**Conclusions:**

The study found that social anxiety can contribute to loneliness by narrowing older adults’ social network. High perceived social support can buffer this process, but do not overstate its protective effects. Thus, interventions to reduce social anxiety and improve social network and social support may help prevent and alleviate loneliness in older adults.

## Background

In the context of rapid population aging, loneliness has become an important topic in public policy and public health. Compared to younger adults, older adults are at a higher risk of social isolation and participate in fewer social activities, which makes them vulnerable to loneliness with declining physical and cognitive abilities, shifting social roles, and worsening social adaptability [1–3]. This subjective emotional experience arises when older adults feel that the quantity and quality of their social relationships do not actually match their expectations [4]. Recently, it was reported that 11.9% of older adults worldwide suffer from loneliness [5]. A study performed by Wei et al. based on data from the 2008/2009 wave from the Chinese Longitudinal Healthy Longevity Survey found that 33.3% of older adults feel lonely [4]. Loneliness is also strongly associated with negative physical and psychological outcomes in older adults. Previous research has shown that higher levels of loneliness may predict frailty, depression, cognitive decline, an increased risk of cardiovascular disease, suicide, and mortality [6–8]. Therefore, it has become a focal point of mental health issues for older adults on how to alleviate loneliness.

People with social anxiety (SA) often exhibit signs of anxiety, fear, or discomfort in social interactions and even avoid social situations to evade negative comments from others [9, 10]. The prevalence of SA often decreases with age. Bai et al.‘s assessment of older persons with chronic disease using the Social Avoidance and Distress Scale revealed a lower score compared to a study of a sample of college students [11]. Nevertheless, the 12-month incidence of diagnosed SA disorders in persons aged 65 and older was reported to be 2.3%, which is the second most frequent anxiety disorder in this age group [12]. Previous studies have demonstrated a substantial correlation between SA and loneliness in later years. For example, a longitudinal study guided by Lim et al. discovered that SA is only significant predictor of future loneliness, with earlier SA positively predicting future loneliness [13]. Hoffman et al. [14] found that older adults with high SA were more likely to experience intimate loneliness (one of the characteristics of loneliness, i.e., lower quantity or quality of intimate companionship) than younger adults. People who feel lonely psychologically actually expect to construct social connections with others [15]. However, avoidance of social situations and concerns about the threat of social situations may prevent these connections from forming, leading to decreased satisfaction with interpersonal relationships, a lack of intimacy, and increased loneliness in older adults [14, 16]. Currently, the association between SA and loneliness has been widely studied in children, adolescents, and young adults [9, 17, 18], while a limited number of studies have been conducted in older adults, most of which use a wide range of ages [13, 19]. Besides, relevant pathways or internal mechanisms between these variables in older adults have not been completely explored, and further investigation is needed to facilitate prevention and interventions for loneliness in older populations.

The interpersonal model of SA proposes that when social situations may trigger SA, people will adopt self-protective strategies that result in low density and dysfunctional social relationships [20]. Whether through active, passive, or a combination of both avoidance strategies, individuals tend to have lower expectations of the outcome of their social interactions [21]. This dysfunctional interpersonal loop has the potential for adverse social effects, such as loneliness [20]. On this basis, in order to establish a more comprehensive model that enhances the understanding of the relationship between SA and loneliness, the study incorporates two variables (social network and perceived social support (PSS)). Social network concerns the number and frequency of relationships a person establishes and maintains (objective structural aspects of social relationships), consisting of family members, close friends, neighbors, and others individuals in their social circle [22, 23]. Previous studies have shown that SA is negatively associated with social network, and those with high SA typically have smaller social networks [24, 25]. The core feature of SA is avoiding social interactions [16], thus anticipating a reduction in the size and frequency of social network in socially anxious individuals. On the other hand, the idea that positive social network is beneficial to an individual’s physical and mental health is widely supported by theoretical and empirical research [26–28]. Loneliness is significantly impacted by social network, with the latter acting as a negative predictor of loneliness [28]. In the Amsterdam Longitudinal Study of Aging, Domènech et al. [29] followed participants over 75 years of age for 11 years and found that decreasing social network size leads to higher levels of loneliness over time. If a person is isolated from family or friends, their perception of quality of life may decline and they are more likely to experience loneliness when functional and emotional needs are not adequately met [30]. Conversely, positive social contacts and wider networks showed the capacity for social adaptation, generating more social resources and support, allowing older adults to maintain a positive attitude and a sense of belonging [31]. Collectively, weak social network was likely to be accompanied by SA and loneliness. Thus, based on the interpersonal model of SA and prior evidence, we hypothesized that SA may exacerbate older adults’ dissatisfaction with family and friend network (loneliness) by reducing the size of these ties and the frequency of contact.

Social support is categorized into received social support and PSS [32]. PSS is broadly defined as individuals’ perceptions of the availability of social support in their networks, emphasizing the subjective emotional experience and satisfaction of an individual when they feel respected, supported and understood in society (functional aspects of social relationships) [23, 32]. Compared to received social support, PSS has a stronger relationship with a person’s mental health [33]. According to Hobfoll’s conservation of resources theory, people are susceptible to resource loss when faced with stressful circumstances, yet protecting and maintaining resources can mitigate the potential negative consequences of stress [34]. Therefore, PSS may act as an adjustable resource to alleviate stress and promote individual mental health. Ren et al. discovered that PSS had a negative association with SA and moderated the link between physical activity and SA in left-behind children [35]. Among older adults, PSS was a protective factor against loneliness, and the predictive effect of chronic diseases on loneliness was more significant with low levels of PSS [36]. However, to our knowledge, no thorough investigation has been made into the mechanisms of the moderating role of PSS in the triadic interaction between SA, social network, and loneliness in older persons. A high level of PSS can make older persons feel valued and motivated to socially interact, expanding their social networks when they suffer anxiety due to socially uncertain situations and negative evaluations. Thus, based on the conservation of resources theory and existing research evidence, we hypothesized that PSS moderates the mediating effect of the social network on the association between SA and loneliness.

One of the most well-liked psychosocial models currently explores how social network and social support buffer the effects of life events or changes on health [22]. Social network highlights the quantity of social relationships, whereas social support emphasizes the function of social relationships. They explain different aspects of interpersonal relationships. By incorporating both social network and PSS into the model, this study aims to provide a more comprehensive understanding of the effects of social relationships on SA and loneliness in older adults, enrich research on loneliness in older adults, and provide a new empirical basis for reducing loneliness in older adults. The research hypothesis are as follows:

### H1

SA is positively associated with loneliness in older adults.

### H2

Social network mediates the relationship between SA and loneliness in older adults.

### H3

PSS moderates the relationship between SA and social network and the relationship between social network and loneliness.

## Methods

### Participants

Using the quota sampling method, a total of 214 older adults in 20 nursing homes of different sizes and 240 older adults living in the community in 5 cities in Zhejiang Province were surveyed from January to May 2021. Inclusion criteria for the elderly in nursing homes comprised the following: (1) age ≥ 60 years; (2) live in a nursing home ≥ 1 month; (3) clear consciousness; and (4) have the ability to read or speak, and communicate with investigators without difficulty. Inclusion criteria for the older adults living in the community were as follows: (1) age ≥ 60 years; (2) choose to age in place; (3) clear consciousness; (4) have the ability to read or speak, and communicate with investigators without difficulty. All older adults combined with serious organic disease or mental illness were excluded.

A priori power analysis with GPower 3.1.9.7 [37] revealed that a linear regression with a significance level of 0.05, power of 0.80, and medium effect size of 0.15 for a maximum of 15 variables (including demographic variables) required a minimum sample size of 139. Besides, studies suggested that sample sizes are generally 5 ~ 10 times the number of variables [38]. Thus, this study yielded a sample size of 150 based on 10 times the number of variables. Considering a 20% attrition rate, the final sample size was estimated to be 174 ~ 188. Ultimately, 454 participants were actually included in the study.

### Data collection

All investigators received standardized training before conducting the investigation, ensuring familiarity with the investigation methods and techniques. During the formal survey, the investigators firstly explained the research objectives and response requirements to older people. Only after obtaining the informed consent did, they distribute the questionnaire on a one-to-one basis. Older adults with literacy skills could fill in the questionnaire by themselves. For illiterate older persons, the investigators would ask questions one by one. If there was any difficulty in comprehension, investigators make neutral and accurate word explanations and record according to their original answers. After completing the questionnaire, a second investigator carefully checked the completeness of the data. Any missing items were filled in promptly and verified on the spot. In this survey, 483 older people were interviewed and they filled out the questionnaires. However, 29 participants did not complete all the items due to missing information or opting out of the program, so they were excluded. Finally, 454 valid questionnaires were acquired for data analysis. The effective response rate of the questionnaire was 93.9%.

## Measures

### Social-demographic information

Socio-demographic data were collected using our own designed short questionnaire, which included age, gender, marriage status, education background, self-reported economic status, degree of filial piety of children, self-rated health and religious belief.

### Social anxiety

The Social Avoidance and Distress Scale (SAD), developed by Watson et al. and translated by Wang et al., was used to assess the SA of the older population [10, 39]. It comprises two dimensions: social avoidance (e.g., “I tend to withdraw from people”) and social distress (e.g., “It’s easy for me to relax when I am with strangers”). The scale has 28 items, 14 of which are rated from 0 (False) to 1 (True), and the remaining 14 items are reverse scored. The total score varied from 0 to 28 with higher scores indicating a higher degree of avoidance or distress. SAD has been validated and used in the older population, although few articles are available [11, 40]. The Cronbach’s α of the scale in this study was 0.90.

### Loneliness

The 6-item De Jong Gierveld Loneliness Scale (DJG-6), developed by deJong Gierveld et al. and translated into Chinese by Leung et al., was applied to assess the level of loneliness [41, 42]. The scale includes two dimensions of emotional loneliness (e.g., “I experience a general sense of emptiness”) and social loneliness (e.g., “There are enough people I feel close to”). Each item has answer categories of no, more or less and yes, where the emotional dimension is 0 (no), more or less (1) and yes (1) and social loneliness dimension is scored using a reverse scoring method. The higher the score, the higher the level of loneliness. The scale has been verified as a tool with high reliability and validity for measuring loneliness in large surveys of older adults [42]. In this study, the Cronbach’s α of the scale is 0.73.

### Social network

The Chinese version of the Lubben Social Network Scale-6 (LSNS-6) was used to measure social network of older adults, demonstrating high reliability and validity [43, 44]. The scale is a 6-item self-report measure with two domains: family network (e.g., “How many relatives do you see or hear from at least once a month?”), and friend network (e.g., “How many of your friends do you see or hear from at least once a month?”). Each item is scored from 0 (none) to 5 (9 or more). The total score ranges from 0 to 30, with a high score indicating a better social network. In this study, the Cronbach’s α was 0.80.

### Perceived social support

The Perceived Social Support Scale (PSSS) was developed by Zimet et al. and revised by Jiang [39, 45]. This scale consists of 12 items organized into 3 dimensions: family support (e.g., “My family can help me concretely”), friend support (e.g., “My friends can share happiness and sadness with me”) and other support (e.g., “Some people (relatives, colleagues, neighbors) in my life care about my feelings”). Each item uses a seven-level scoring method from 1(totally disagree) to 7 (completely agree). The total score ranges from 12 to 84, and a higher score indicates a higher level of PSS. The Cronbach’s α in this study was 0.91.

### Ethical considerations

This study was approved by the Institutional Review Board of Wenzhou Medical University (approval number: 2021-011). First, the principle of informed consent was abided by before administering the survey. Second, the identity information of the participants was kept strictly confidential and not disclosed to members outside the research team. Third, Participants had the right to decide whether to participate or not, and can withdraw from the investigation at any time. All methods were performed in accordance with the relevant rules and regulations of the Declaration of Helsinki.

### Data analysis

SPSS 26.0 was applied for statistical analysis. The numeric data in this study were identified as non-normal distribution after the Kolmogorov-Smirnov test, so the non-normal distributed data were described by the median and interquartile range. Categorical data were expressed in frequency and percentage. The Mann-Whitney U test and the Kruskal-Wallis test were used to assess differences in loneliness across demographic characteristics. Spearman correlation was used for correlation analysis between variables. Considering that the children’s filial piety and self-rated health status may be related to loneliness, they were included as control variables [46, 47]. Model 4 and 58 in the SPSS 26.0 macros program PROCESS compiled by Hayes [48], was used to construct the moderated mediation model with 5,000 bootstrap sampling. To further explore the moderating effect of PSS, PSS was divided into two groups, namely, high and low PSS by adding and subtracting a standard deviation by mean. Subsequently, the simple slope analysis was carried out. P value < 0.05 was considered statistically significant. To check for the possibility of common method deviation, Harman’s single factor test was used. It is based on exploratory factor analysis to determine the number of factors needed to explain the variance of a variable. The bias is more likely if a single factor precipitates or explains a greater variance [49]. The variance explained by a single factor is generally considered to be less than 40% [50].

## Results

### Common method bias

The results showed that 12 factors with eigenvalue greater than 1 were co-precipitated, and the variance explained by the first factor was 20.03%, which was less than the critical standard of 40%, indicating that the common method deviation of this study was not significant.

### Demographic characteristics

Table 1 lists the demographic characteristics. The average age of the 454 older adults (214 older people in nursing homes and 240 in the community) was 76.3 ± 8.5 years old, with 193 (42.5%) people aged 60 ~ 74 years and 235 (51.8%) people aged 75 ~ 89 years. 204 (55.1%) were women and 232 (51.1%) were married. In terms of educational background, only 31.3% of the participants graduated from junior high school or above. A total of 395 participants (87%) self-reported their economic status was roughly enough and enough and excess. Most participants (77.1%)perceived their children to be filial. Furthermore, 55.9% of the elderly rated their health as good and 39.4% had no religious beliefs. Comparative analyses of loneliness based on demographic characteristics showed no statistically significant differences in loneliness scores with respect to age, gender, marital status, education background, self-reported economic status and religious belief. Regarding the degree of filial piety of their children, loneliness scores were lower among older adults who perceived their children to be filial. Besides, older adults with good self-rated health had lower loneliness scores compared to those with poorer self-rated health.

**Table 1:** Social-demographic characteristics and comparison of loneliness scores in different groups (*n* = 454)

Variable	N (%)	Loneliness (median (IQR))	Z/H
**Age (years)**			
60 ~ 74	193 (42.5)	3.00 (4.00)	0.66
75 ~ 89	235 (51.8)	3.00 (4.00)	
≥ 90	26 (5.7)	2.00 (3.00)	
**Gender**			
Male	204 (44.9)	3.00 (4.00)	−1.71
Female	250 (55.1)	3.00 (3.00)	
**Marital status**			
Married	232 (51.1)	2.00 (3.00)	−1.81
Single/divorced/widowed	222 (48.9)	3.00 (4.00)	
**Education background**			
Illiterate	138 (30.4)	3.00 (3.00)	1.42
Graduated primary school	174 (38.3)	3.00 (3.25)	
Graduated junior high school and above	142 (31.3)	3.00 (4.00)	
**Self-reported economic status**			
Enough and excess	221 (48.7)	3.00 (4.00)	4.85
Roughly enough	174 (38.3)	3.00 (4.00)	
difficulties	59 (13.0)	2.00 (3.00)	
**Degree of filial piety of children**			
Filial	350 (77.1)	2.00 (3.00)	54.53***
General/unfilial	88 (19.4)	5.00 (1.25)	
Childless	16 (3.5)	3.00 (3.75)	
**Self-rated health**			
Good	254 (55.9)	2.00 (3.00)	33.30***
Moderate	141 (31.1)	4.00 (3.00)	
Poor	59 (13.0)	4.00 (3.00)	
**Religious belief**			
Strong	103 (22.7)	3.00 (4.00)	0.17
General	172 (37.9)	3.00 (4.00)	
No	179 (39.4)	3.00 (4.00)	

*Abbreviations*: IQR: Interquartile Range

*Note*: ***P < 0.001

### Correlations among the main variables

In this study, the correlations among the four variables of SA, social network, loneliness and PSS were analyzed. The correlation matrix was presented in Table 2. The results showed that: (1) SA was significantly negatively correlated with social network and PSS, but significantly positively correlated with loneliness; (2) Social network was significantly negatively correlated with loneliness, but significantly positively correlated with PSS; (3) Loneliness was significantly negatively correlated with PSS. All data were standardized before further analysis.

**Table 2:** Correlation coefficient of SA, social network, loneliness and PSS

	Median (IQR)	1. SA	2. Social network	3. Loneliness	4. PSS
1. SA	7.00 (10.25)	1			
2. Social network	15.00 (10.00)	− 0.292**	1		
3. Loneliness	3.00 (4.00)	0.484**	− 0.416**	1	
4. PSS	64.00 (18.00)	− 0.298**	0.558**	− 0.524**	1

*Abbreviations*: IQR: Interquartile Range; SA: Social anxiety; PSS: Perceived social support

*Note*: **p < 0.01. Spearman correlation was used for correlation analysis

### Mediation model

As shown in Table 3, after controlling for filial piety and self-rated health, SA was positively related to loneliness (*Β* = 0.40, t = 9.68, *P* < 0.001). After adding social network as a mediating variable, SA (*Β* = 0.33, t = 8.07, *P* < 0.001) and social network (*Β* = −0.27, t = −6.80, *P* < 0.001) were positively and negatively correlated with loneliness, respectively. Besides, SA was found to significantly negatively predict social network (*Β* = −0.26, t = −5.62, *P* < 0.001). In the bias-corrected percentile bootstrap analysis, the mediating effect of social network on the relationship between SA and loneliness was significant (ab = 0.07, 95% CI [0.04, 0.11]), accounting for 17.74% of the total effect. Regarding the direct effect of SA on loneliness, the 95% CI ([0.25, 0.41]) did not contain 0, indicating social network partially mediated the relationship between SA and loneliness.

**Table 3:** Testing the mediation effects of social network in the relation between SA and loneliness

Regression equation		Global fit index			Significance of regression coefficient	
Outcome variable	Predictor variable	R	R^2^	F	Β (95% CI)	t
Loneliness	Filial piety	0.53	0.28	58.01	0.43 (0.28, 0.59)	5.53***
	Self-rated health				0.13 (0.04, 0.22)	2.92**
	SA				0.40 (0.32, 0.49)	9.68***
Social network	Filial piety	0.33	0.11	18.59	−0.32 (−0.50, −0.15)	−3.74***
	Self-rated health				0.03 (−0.12, 0.07)	0.51
	SA				−0.26 (−0.35, −0.17)	−5.62***
Loneliness	Filial piety	0.59	0.35	59.44	0.34 (0.19, 0.49)	4.53***
	Self-rated health				0.12 (0.04, 0.21)	2.90**
	SA				0.33 (0.25, 0.41)	8.07***
	Social network				−0.27 (−0.35, −0.20)	−6.80***

*Abbreviations*: SA: Social anxiety

*Note*: **P < 0.01, ***P < 0.001. Filial piety and self-rated health were analyzed as control variable

### Moderated mediation model

The moderated mediation model testing result was displayed in Table 4. SA has a significant negative predictive effect on social network (*Β* = −0.15, t = −3.59, *P* < 0.001). SA (*Β* = 0.31, t = 7.89, *P* < 0.001) and social network (*Β* = −0.10, t = −2.26, *P* < 0.05) were positively and negatively related to loneliness, respectively. PSS was significantly negatively predicted loneliness (*Β* = −0.35, t = −7.28, *P* < 0.001). The interaction effect of SA and PSS on social network was significant (*Β* = −0.08, t = −2.13, *P* < 0.05), indicating that the pathway of “SA → social network” was moderated by PSS. Moreover, the interaction effect of social network and PSS on loneliness was significant (*Β* = −0.11, t = −3.07, *P* < 0.01) indicating that the pathway of “social network → loneliness” was moderated by PSS.

**Table 4:** Testing the moderated mediation effect in the relation between SA and loneliness

Regression equation		Global fit index			Significance of regression coefficient	
Outcome variable	Predictor variable	R	R^2^	F	Β (95% CI)	t
Social network	Filial piety	0.59	0.35	47.42	−0.01 (−0.16, 0.15)	−0.12
	Self-rated health				0.02 (−0.06, 0.11)	0.55
	SA				−0.15 (−0.23, −0.07)	−3.59***
	PSS				0.55 (0.46, 0.63)	12.70***
	SA×PSS				−0.08 (−0.16, −0.01)	−2.13*
Loneliness	Filial piety	0.65	0.42	53.25	0.25 (0.10, 0.39)	3.25**
	Self-rated health				0.08 (0.00, 0.16)	1.85
	SA				0.31 (0.23, 0.39)	7.89***
	Social network				−0.10 (−0.19, −0.01)	−2.26*
	PSS				−0.35 (−0.45, −0.26)	−7.28***
	Social network × PSS				−0.11 (−0.18, −0.04)	−3.07**

*Abbreviations*: SA: Social anxiety; PSS: Perceived social support

*Note*: *P < 0.05, **P < 0.01, ***P < 0.001. Filial piety and self-rated health were analyzed as control variable

**Fig. 1:**
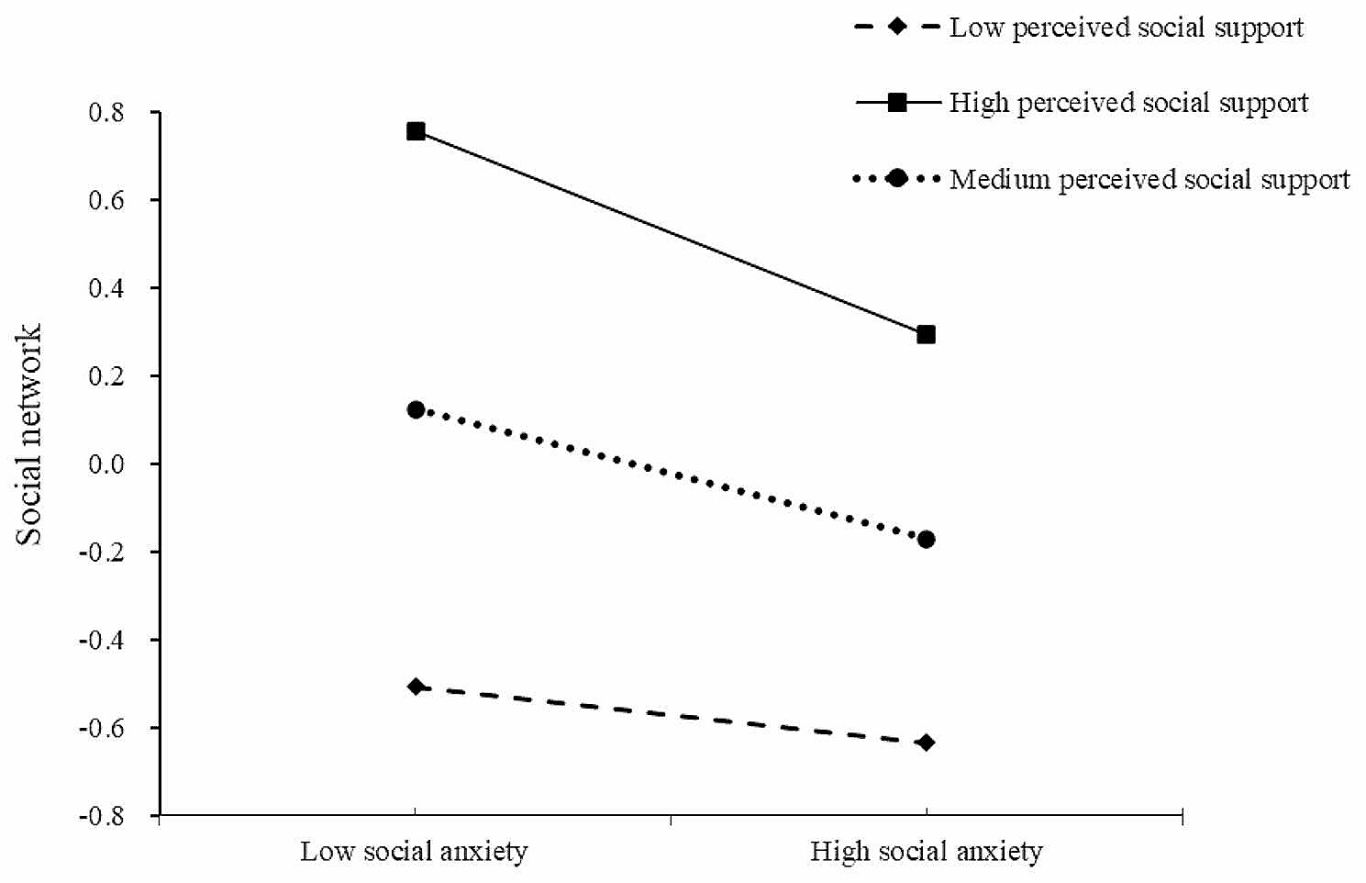
The moderating effect of perceived social support on social anxiety and social network

To further analyze the moderating effect of PSS in the mediation model, we regarded the mean of PSS plus one standard deviation (M + SD) as the high-level group, the mean (M) as the medium-level group, and the mean minus one standard deviation (M-SD) as the low-level group for simple slope analysis. The moderating effect of different levels of PSS between SA and social network was shown in Table 5 and Fig. 1. The results revealed that the predictive effect of SA on the social network of the elderly with medium PSS (M) was lower than that of the elderly with high PSS (*Β*_medium PSS_ = −0.15, *p* < 0.001; *Β*_high PSS_ = −0.23, *p* < 0.001), whereas the relationship between SA and loneliness was not found in low PSS (*Β*_low PSS_ = −0.06, *p* > 0.05). It indicated that the predictive effect of SA on social network increased gradually with the improvement of PSS of the elderly.

**Table 5:** The moderating effect of different levels of PSS between SA and social network

PSS	Effect size	SE	Boot LLCI	Boot ULCI
M-SD	−0.06	0.06	−0.17	0.05
M	−0.15***	0.04	−0.23	−0.07
M + SD	−0.23***	0.06	−0.34	−0.12

*Abbreviations*: SA: Social anxiety; PSS: Perceived social support; LL: Low Limit; UL: Upper Limit

*Note*: ***P < 0.001

**Fig. 2:**
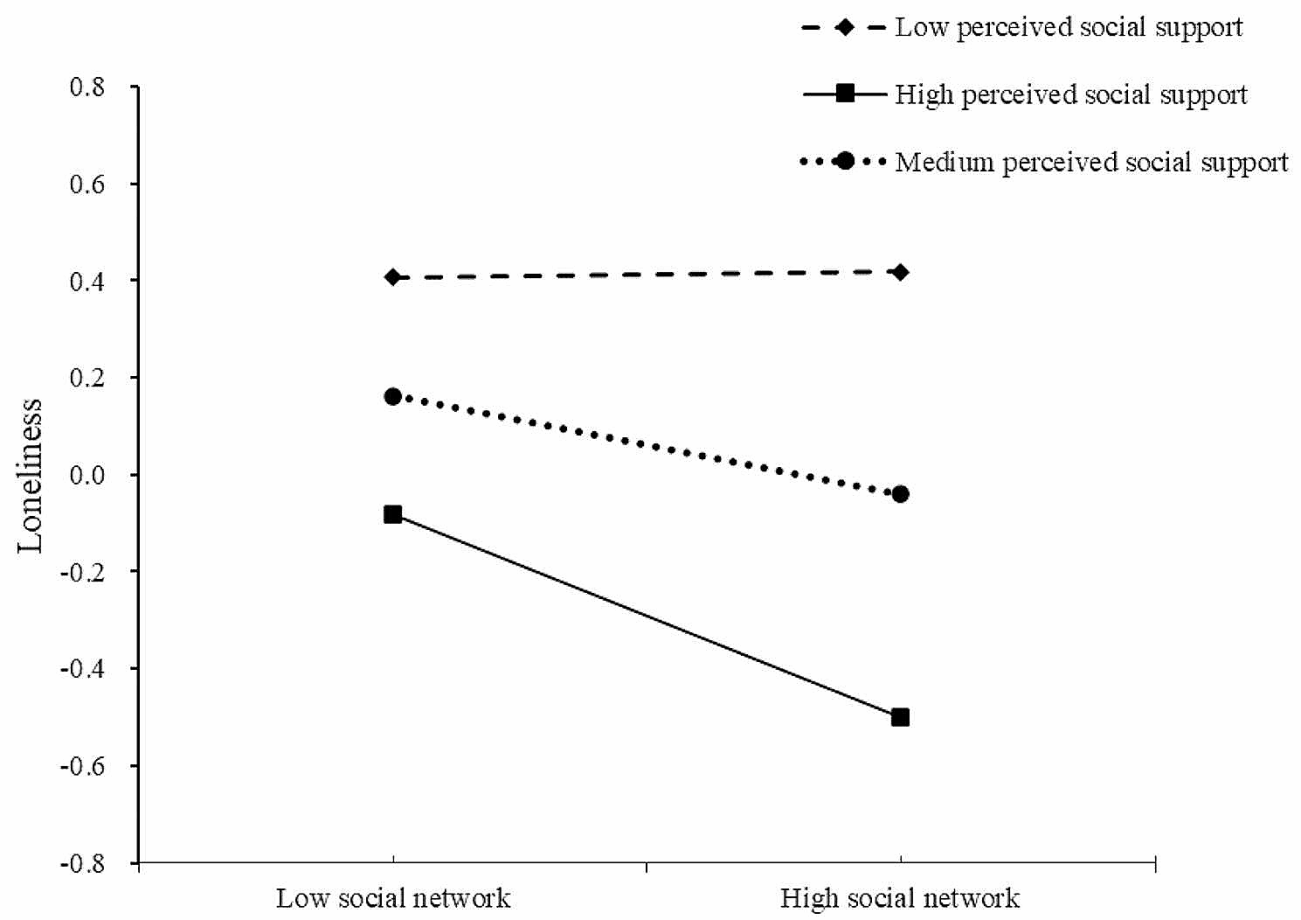
The moderating effect of perceived social support on social network and loneliness

The moderating effect of different levels of PSS between social network and loneliness was shown in Table 6 and Fig. 2. The results revealed that social network of older adults with medium PSS were less effective in predicting loneliness than those with high PSS (*Β*_medium PSS_ = −0.10, *p* < 0.05; *Β*_high PSS_ = −0.21, *p* < 0.001), whereas the relationship between social network and loneliness was not found in low PSS (*Β*_low PSS_ = 0.01, *p* > 0.05). This showed that the predictive effect of social network on loneliness gradually increased with the improvement of PSS for older adults.

**Table 6:** The moderating effect of different levels of PSS between social network and loneliness

PSS	Effect size	SE	Boot LLCI	Boot ULCI
M-SD	0.01	0.06	−0.11	0.13
M	−0.10*	0.05	−0.19	−0.01
M + SD	−0.21***	0.05	−0.31	−0.11

*Abbreviations*: PSS: Perceived social support; LL: Low Limit; UL: Upper Limit

*Note*: *P < 0.05, ***P < 0.001

## Discussion

This study employed social network as the mediating variable and PSS as the moderating variable to construct a moderated mediation model. In addition to elucidating the processes by which SA affects loneliness in older adults, this novel evidence provided important responses to the conditions under which SA affects loneliness, and contributed to the development of effective targeted interventions. Specifically, SA had an impact on older adults’ loneliness through social network. Additionally, PSS moderated the relationship between SA and social network as well as the relationship between social network and loneliness.

### The mediating role of social network

In this study, SA had a significant positive predictive effect on loneliness, in line with findings from several studies [14, 19]. The outcome highlights SA is a risk factor of becoming lonely, and that assessing and intervening in SA in older adults can help identify and reduce loneliness. Furthermore, social network was further found to play a mediating role between SA and loneliness, confirming the validity of hypothesis 2. SA not only affected feelings of loneliness directly, but also exerted an indirect effect on loneliness through social network. Several reasons contribute to this phenomenon: first, over time, older adults may experience negative events, such as age-related physical limitation, retirement or widowhood, which lead to negative self-perceptions and feelings of disconnection from an increasingly fast-paced and rapidly evolving society [51, 52]. Experience SA, especially if fearful of social situations and negative evaluations, can lead to small network and infrequent social interaction, making it more difficult to build and sustain intimate social ties [24, 25]. Consequently, they are more susceptible to social isolation. Second, studies have consistently shown a strongly negative relationship between social network and loneliness [15, 28, 53]. Social network can serve as a protective factor against loneliness when they satisfy older adults’ demands for a sense of belonging and desired social relationships [53]. Due to the influence of the traditional culture of filial piety in China [4], most elderly have deep-rooted family values and expect close, interdependent family relationships. However, as children move out and the proportion of empty nesters gradually increases, the size and density of older adults’ family network may gradually decrease [27]. The social network will be single and fixed. Many senior citizens choose to migrate to their adult children’s hometowns, accompanied by language and cultural barriers, may contribute to a decline in existing network, making it challenging to make new friends [51, 54]. All these problems can lead to social isolation and loneliness.

Altogether, the study of the mediating role of social network has enriched the understanding of the inter mechanisms underlying the relationship between SA and loneliness, highlighted the exploration of the antecedents of social network, and validated the interpersonal model of SA. Future practice should pay attention to developing effective strategies that not only address the issue of SA among older people, but also strive to strengthen social network in order to reduce the occurrence of loneliness.

### The moderating role of PSS

According to hypothesis 3, the results demonstrated that PSS moderated the mediation role of social network. In the pathway of “SA → social network”, social network was higher in the high PSS state at both low and high SA, suggesting a protective effect of PSS on social network reduction due to SA. However, this protective effect rapidly diminished with higher SA, indicating that PSS is a stress-vulnerability factor in pathway 1. Although there was no support for stress-buffering in the result, there was also an indication of the protective effect of PSS. Rueger et al.’s study on the association between PSS and depression conceptualized protective factors’ buffering effect in two different ways: stress-buffering (effects of social support are enhanced) and reverse stress-buffering (effects of social support are dampened) [55]. The finding of this study was consistent with reverse stress-buffering model, where the protective effect of PSS is limited. High level of PSS arose from interpersonal relationships, which, to some extent, created part of the social network for older adults. It can boost confidence in older adults and keep them emotionally upbeat to cope with stressful threats in social settings, therefore becoming useful in preventing the reduction of social network in older adults suffering from SA [3, 56]. The cognitive-behavioral model of SA [57], however, posits that individuals with SA will be preoccupied with self-representations that they regard as faulty or related to not meeting social expectations or norms. When seeking PSS, older adults may focus on diminished physical functioning, compare themselves to those believed to be highly capable, and acquire low self-esteem [58, 59]. On the other hand, when older adults suffer from high level SA, they may be more sensitive to any perceived differences in social relationships and adopt negative and avoiding attitudes, thereby denying appropriately received social support and resulting in a lack of PSS. Low self-esteem and hypersensitivity to social situations weakened the protective role of PSS in the process of SA-induced social network narrowing. It might also be understood that once stress reaches a certain level, PSS may lose its ability to offset SA. In the case of low PSS, older adults’ social networks may have been restricted. Therefore, their social networks changed less regardless of high or low SA.

Regarding the pathway of “social network → loneliness”, the study also found that loneliness decreased significantly with the increase of social network in the case of high PSS, suggesting that PSS plays a facilitating role in the process of social network affecting loneliness, consistent with the conservation of resources theory [34]. When older adults feel high PSS, they gain more value-affirming, self-worth and life satisfaction [3, 30]. They exhibit more positivity and optimism when interacting with others and are able to perceive the benefits of objective social networks, which, in turn considerably improve social networks, such as network size [2, 29], and reduce loneliness. In addition, the connection between older adults and those in their social networks who provide support not only promotes the development of intimate relationships, but also increases the likelihood of exposure to health information, further boosting mental health [60]. Conversely, the study discovered that in the event of reported poor PSS, the decrease in loneliness did not change significantly with increasing social network, indicating that the elderly could not effectively access the positive effect of social network when lacking social support. Therefore, they were unable to successfully reduce loneliness through expanding their social network. Accordingly, building a strong social support network is an important way for older adults to maintain a good psychological state since it may help social network strengthen and mitigate loneliness. Nevertheless, given the relatively limited protective effect of PSS, just trying to increase their level of PSS may not be appropriate for lonely older adults experiencing SA. How to reduce SA in older adults may be the focus of interventions.

### Limitations

The current research still has some limitations. First, as the study was designed as a cross-sectional survey, making causal inferences was not possible. However, the moderated mediation model was based on a theoretical foundation and supported by previous empirical studies, so the cross-sectional survey can still provide valuable information about the relationships between variables. More longitudinal studies are needed in the future to improve the representativeness of the moderated mediation model. Second, the LSNS- 6 scale was used to measure social network in the study, which includes relatively objective items characterizing the structure of social relationships (i.e. size of active network, size of intimate network and frequency of contact). But the size of social network and the frequency of contact among different older adults might affect the availability and effectiveness of resources as well as the study results. It is recommended that the role of these variables be further explored to suggest more detailed interventions. Third, although the questionnaire used in this study has been validated in previous studies with satisfactory reliability and validity, all variables were based on older adults’ self-report, introducing the possibility of self-report bias. Finally, due to the dialect problem, only non-random sampling, rather than random sampling, could be used in the study, which affected the representativeness of the study sample to some extent.

### Relevance to clinical practice

This study provides new perspectives for nursing home or community managers to lessen loneliness in older adults. First, prompt screening and treatment for SA can be an effective measure to identify and reduce loneliness in older adults. The degeneration of psychological and physical functions brought about by aging makes the elderly less adaptable to social and more fearful of negative evaluation, so they tend to avoid social situations. It is necessary to provide cognitive behavioral training to this population to reduce SA. Second, older people with SA may not initiate social interactions. Social assistance and inviting them to participate in community building or in the daily management of the nursing home contribute to the expansion of social network. Third, the assessment and intervention of PSS also deserve attention. Measures to increase the perceived support of older adults, especially emotional support from family and friends, should be taken along with improving social network to enhance their use of PSS and reduce loneliness. For example, nursing home or community managers can work with family and friends to establish small social support groups, organizing regular speech contests, group interaction activities, and changing the location of the event from time to time to help participants with SA adjust to different social situations. However, it’s crucial not to overstate the role of protective resources such as PSS.

## Conclusion

Collectively, although further in-depth research is needed, this research represents an important step in clarifying the relationship between SA and loneliness among elderly. The results addressed the question of how SA affects the loneliness in older adults, highlighting the negative predictive effect of SA on loneliness and identifying conditions under which the mediating effect of social network and the moderating effect of PSS are more significant. These findings emphasized the importance of psychosocial factors and revealed the potential mechanisms by which SA affects loneliness in older adults. Additionally, it can assist nursing home or community managers in carrying out efficient psychological interventions to further improve the quality of life and psychological well-being of older adults, meeting the emotional needs of society.

## Data Availability

The data that support the findings of this study are available from the corresponding author, upon reasonable request.

## Abbreviations

SA: Social Anxiety
PSS: Perceived Social Support
SAD: The Social Avoidance and Distress Scale
DJG-6: The 6-item De Jong Gierveld Loneliness Scale
LSNS-6: The Lubben Social Network Scale-6
PSSS: The Perceived Social Support Scale

## References

[1] Barakat MM, Elattar NF, Zaki HN, Depression (2019). Anxiety and loneliness among Elderly Living in Geriatric homes. Am J Nurs Res.

[2] Kemperman A, van den Berg P, Weijs-Perrée M, Uijtdewillegen K. Loneliness of older adults: Social Network and the living environment. Int J Environ Res Public Health. 2019;16(3). 10.3390/ijerph16030406. 10.3390/ijerph16030406 PMC6388289 30708985

[3] Jia Y, Yue Y (2023). Perceived social support mediates loneliness and social isolation: a cross-sectional study of Chinese older adults relocated for poverty relief. Int J Geriatr Psychiatry.

[4] Wei K, Liu Y, Yang J, Gu N, Cao X, Zhao X (2022). Living arrangement modifies the associations of loneliness with adverse health outcomes in older adults: evidence from the CLHLS. BMC Geriatr.

[5] Surkalim DL, Luo M, Eres R, Gebel K, van Buskirk J, Bauman A (2022). The prevalence of loneliness across 113 countries: systematic review and meta-analysis. BMJ.

[6] Cacioppo S, Grippo AJ, London S, Goossens L, Cacioppo JT (2015). Loneliness: clinical import and interventions. Perspect Psychol Sci.

[7] Cacioppo JT, Hawkley LC (2009). Perceived social isolation and cognition. Trends Cogn Sci.

[8] Gale CR, Westbury L, Cooper C (2018). Social isolation and loneliness as risk factors for the progression of frailty: the English Longitudinal Study of Ageing. Age Ageing.

[9] Maes M, Nelemans SA, Danneel S, Fernández-Castilla B, Van den Noortgate W, Goossens L (2019). Loneliness and social anxiety across childhood and adolescence: Multilevel meta-analyses of cross-sectional and longitudinal associations. Dev Psychol.

[10] Watson D, Friend R (1969). Measurement of social-evaluative anxiety. J Consult Clin Psychol.

[11] Bai JW, Hao XJ, li CZ, Cheng SC (2021). Social avoidance and distress among middle-aged and older adults with chronic diseases and influencing factors. Chin J Gerontol.

[12] Gum AM, King-Kallimanis B, Kohn R (2009). Prevalence of mood, anxiety, and substance-abuse disorders for older americans in the national comorbidity survey-replication. Am J Geriatr Psychiatry.

[13] Lim MH, Rodebaugh TL, Zyphur MJ, Gleeson JF (2016). Loneliness over time: the crucial role of social anxiety. J Abnorm Psychol.

[14] Hoffman YSG, Grossman ES, Bergman YS, Bodner E (2021). The link between social anxiety and intimate loneliness is stronger for older adults than for younger adults. Aging Ment Health.

[15] Cacioppo JT, Cacioppo S. Chapter three - loneliness in the modern age: an evolutionary theory of loneliness (ETL). In: Olson JM, editor. Advances in experimental social psychology. Volume 58. Academic Press; 2018. pp. 127–97.

[16] Lieberz J, Shamay-Tsoory SG, Saporta N, Kanterman A, Gorni J, Esser T (2022). Behavioral and neural dissociation of social anxiety and loneliness. J Neurosci.

[17] Chau AKC, So SH, Sun X, Zhu C, Chiu CD, Chan RCK (2022). The co-occurrence of multidimensional loneliness with depression, social anxiety and paranoia in non-clinical young adults: a latent profile analysis. Front Psychiatry.

[18] Danneel S, Nelemans S, Spithoven A, Bastin M, Bijttebier P, Colpin H (2019). Internalizing problems in adolescence: linking loneliness, social anxiety symptoms, and depressive symptoms over Time. J Abnorm Child Psychol.

[19] Bruce LD, Wu JS, Lustig SL, Russell DW, Nemecek DA (2019). Loneliness in the United States: a 2018 National Panel Survey of demographic, structural, cognitive, and behavioral characteristics. Am J Health Promot.

[20] Alden LE, Taylor CT (2004). Interpersonal processes in social phobia. Clin Psychol Rev.

[21] Kim SSY, Liu M, Qiao A, Miller LC (2022). I want to be alone, but I don’t want to be lonely: uncertainty management regarding social situations among College students with social anxiety disorder. Health Commun.

[22] Berkman LF (1983). The assessment of social networks and social support in the elderly. J Am Geriatr Soc.

[23] Donev D, Pavlekovic G, Zaletel-Kragelj L. Social Networks and Social Support in Health Promotion Programmes. 2008. 10.2390/biecoll-mhcp4-1.7.

[24] Falk Dahl CA, Dahl AA (2010). Lifestyle and social network in individuals with high level of social phobia/anxiety symptoms: a community-based study. Soc Psychiatry Psychiatr Epidemiol.

[25] Teo AR, Lerrigo R, Rogers MA (2013). The role of social isolation in social anxiety disorder: a systematic review and meta-analysis. J Anxiety Disord.

[26] Berkman LF, Glass T, Brissette I, Seeman TE (2000). From social integration to health: Durkheim in the new millennium. Soc Sci Med.

[27] Chang H, Zhou J, Wang Z (2022). The impact of social capital on successful ageing of empty nesters: a cross-sectional study. J Adv Nurs.

[28] Santini ZI, Jose PE, York Cornwell E, Koyanagi A, Nielsen L, Hinrichsen C (2020). Social disconnectedness, perceived isolation, and symptoms of depression and anxiety among older americans (NSHAP): a longitudinal mediation analysis. Lancet Public Health.

[29] Domènech-Abella J, Mundó J, Switsers L, van Tilburg T, Fernández D, Aznar-Lou I (2021). Social network size, loneliness, physical functioning and depressive symptoms among older adults: examining reciprocal associations in four waves of the Longitudinal Aging Study Amsterdam (LASA). Int J Geriatr Psychiatry.

[30] Moreno-Tamayo K, Manrique-Espinoza B, Ramírez-García E, Sánchez-García S (2020). Social isolation undermines quality of life in older adults. Int Psychogeriatr.

[31] Zhao D, Zhao M, Wang N, Fu M, Wang A (2020). Relationships among social isolation, depression, loneliness and quality of life in the community-dewelling elderly. J Nurs Sci.

[32] Xiao S, Yang D (1987). The effects of social support on physical and mental health. Chin Mental Health J.

[33] Liu Y, Aungsuroch Y (2019). Work stress, perceived social support, self-efficacy and burnout among Chinese registered nurses. J Nurs Manag.

[34] Hobfoll SE (2001). The influence of culture, community, and the nested-self in the stress process: advancing conservation of resources theory. Appl Psychology: Int Rev.

[35] Ren Y, Li M (2020). Influence of physical exercise on social anxiety of left-behind children in rural areas in China: the mediator and moderator role of perceived social support. J Affect Disord.

[36] Xiao SJ, Shi L, Dong F, Zhang JC, Xue BL, Ouyang P (2021). The impact of chronic diseases on loneliness among the older adults: the mediating effect of cognitive function and moderating role of perceived social support. Mod Prev Med.

[37] Faul F, Erdfelder E, Lang AG, Buchner A (2007). G*Power 3: a flexible statistical power analysis program for the social, behavioral, and biomedical sciences. Behav Res Methods.

[38] Tinsley HEA, Tinsley DJ (1987). Uses of factor analysis in counseling psychology research. J Couns Psychol.

[39] Wang XD, Wang XL, Ma H (1999). Rating scales for mental health (supplement).

[40] Liu YR. Research on the involvement of group work in the community integration of old drifters [master]. Dalian Maritime University; 2020.

[41] Gierveld JDJ, Tilburg TV (2006). A 6-Item scale for overall, emotional, and Social Loneliness:confirmatory tests on Survey Data. Res Aging.

[42] Leung GT, de Jong Gierveld J, Lam LC (2008). Validation of the Chinese translation of the 6-item De Jong Gierveld loneliness scale in elderly Chinese. Int Psychogeriatr.

[43] Lubben J, Blozik E, Gillmann G, Iliffe S, von Renteln Kruse W, Beck JC (2006). Performance of an abbreviated version of the Lubben Social Network Scale among three European community-dwelling older adult populations. Gerontologist.

[44] Chang Q, Sha F, Chan CH, Yip PSF (2018). Validation of an abbreviated version of the Lubben Social Network Scale (LSNS-6) and its associations with suicidality among older adults in China. PLoS ONE.

[45] Zimet GD, Powell SS, Farley GK, Werkman S, Berkoff KA (1990). Psychometric characteristics of the Multidimensional Scale of Perceived Social Support. J Pers Assess.

[46] Zheng X, Li H (2022). How Chinese children’s filial piety beliefs affect their parents’ life satisfaction and loneliness. Psych J.

[47] Sol K, Brauer S, Antonucci TC (2023). Longitudinal associations between loneliness and self-rated Health among Black and White older adults. J Gerontol B Psychol Sci Soc Sci.

[48] Hayes AF. Introduction to mediation, moderation, and conditional process analysis: A regression-based approach. New York, NY, US: Guilford Press; 2013. xvii, 507-xvii, p.

[49] Podsakoff PM, MacKenzie SB, Lee JY, Podsakoff NP (2003). Common method biases in behavioral research: a critical review of the literature and recommended remedies. J Appl Psychol.

[50] Tang DD, Wen ZL (2020). Statistical approaches for testing common method bias: problems and suggestions. J Psychol Sci.

[51] Singh A, Misra N (2009). Loneliness, depression and sociability in old age. Ind Psychiatry J.

[52] Fu TT, Zhang QZ, Shen CZ (2019). Meta-synthesis of qualitative studies on the elderly’s experience towards self-perception of aging. Military Nurs.

[53] de Jong Gierveld J, van Tilburg T, Dykstra PA, Vangelisti AL, Perlman D (2006). Loneliness and social isolation. The Cambridge Handbook of Personal Relationships. Cambridge Handbooks in psychology.

[54] Luo EL, Mei SW, Wu KR, Wu YF, Jin T. A study on the construction model of the new social network of elderly urban immigrants. Urban Insight. 2023;01119–31. 10.3969/j.issn.1674-7178.2023.01.011.

[55] Rueger SY, Malecki CK, Pyun Y, Aycock C, Coyle S (2016). A meta-analytic review of the association between perceived social support and depression in childhood and adolescence. Psychol Bull.

[56] Liu XY, Jin YC, An JX (2020). Social support and loneliness in young adults: a meoderated mediation model. J Psychol Sci.

[57] Rapee RM, Heimberg RG (1997). A cognitive-behavioral model of anxiety in social phobia. Behav Res Ther.

[58] Goodman FR, Kelso KC, Wiernik BM, Kashdan TB (2021). Social comparisons and social anxiety in daily life: an experience-sampling approach. J Abnorm Psychol.

[59] Huang WJ, Fu GS, Tan LN, Li CZ, Wei QL, Gao Q et al. Self-esteem and life satisfaction of urban empty nesters and influencing factors. Chinese Journal of Gerontology. 2021;41(06):1326-9. https://doi.org/3969/j.issn.1005-9202.2021.06.056

[60] Henry JD, Coundouris SP, Mead J, Thompson B, Hubbard RE, Grainger SA. Social Frailty in Late Adulthood: Social Cognitive and Psychological Well-Being correlates. J Gerontol B Psychol Sci Soc Sci. 2023;78(1):87–96. 10.1093/geronb/gbac157. 10.1093/geronb/gbac157 PMC9890915 36190802

